# Phenotyping Antibody-mediated Rejection Without Detectable Donor-specific Antibodies 8 d After Heart Transplantation: A Case Study

**DOI:** 10.1097/TXD.0000000000002016

**Published:** 2026-10-21

**Authors:** Sarah Flint, Aiwen Zhang, Gabriel Kim, Amanda Vest, Miriam Jacob, Marc Halushka, Paulino Alvarez

**Affiliations:** 1 Section of Heart Failure and Cardiac Transplantation, Department of Cardiovascular Medicine, Cleveland Clinic, Cleveland, OH.; 2 Department of Pathology and Laboratory Medicine, Histocompatibility and Immunogenetics, Cleveland Clinic, Cleveland, OH.; 3 Department of Pathology and Laboratory Medicine, Cardiovascular Pathology, Cleveland Clinic, Cleveland, OH.

## INTRODUCTION

Early antibody-mediated rejection (AMR) within the first month after transplantation is typically associated with preexisting or de novo HLA donor-specific antibodies (DSAs). We report a case of AMR occurring 8 d after heart transplantation without detectable DSAs and describe the potential utility of gene expression profiling and non-HLA antibody testing, along with clinical evaluation, for risk stratification and treatment guidance.

## CASE PRESENTATION

A 53-y-old African American male had a HeartWare left ventricular assist device (LVAD) as a bridge to transplant for 6 y. He developed late pump thrombosis requiring exchange and chronic Pseudomonas aeruginosa driveline infection. Ultimately, he was admitted for evaluation of recurrent controller fault alarms and underwent heart transplantation 73 d later.

His pretransplant calculated panel reactive antibody was 34% (**Table S1**, **SDC**, https://links.lww.com/TXD/A916), and the virtual crossmatch was negative. Cold ischemic time was 3 h 30 min. A reusable, iceless cooler was the cold storage method used in this case. Postoperatively, he was treated with corticosteroids, mycophenolate mofetil, and induction therapy with basiliximab because of stage 3 chronic kidney disease. Postoperative complications included the requirement for continuous renal replacement therapy. Tacrolimus was initiated on postoperative day (POD) 3. Retrospective flow cytometry crossmatch was negative for both B and T cells.

On POD 8, his first endomyocardial biopsy (EMB) revealed AMR with 3+ C4d and 3+ C3d (Figure 1A–C). There was no evidence of microvascular inflammation in the specimens reviewed. In conjunction, these findings are consistent with the International Society for Heart and Lung Transplantation classification of pathologic AMR, specifically immunopathologic pAMR 1 (I+). A repeat biopsy was performed on POD 10, which showed acute cellular rejection (ACR) 2R, 2+ C3d, and 3+ C4d. The tacrolimus level at that time was 4.1 ng/mL. Right heart catheterization showed a right atrial pressure of 7 mm Hg, right ventricular pressure of 36/7 mm Hg, pulmonary arterial pressure of 36/12 mm Hg, pulmonary capillary wedge pressure of 5 mm Hg, and cardiac index by thermodilution of 2.01 L/min/m^2^.

**FIGURE 1.**
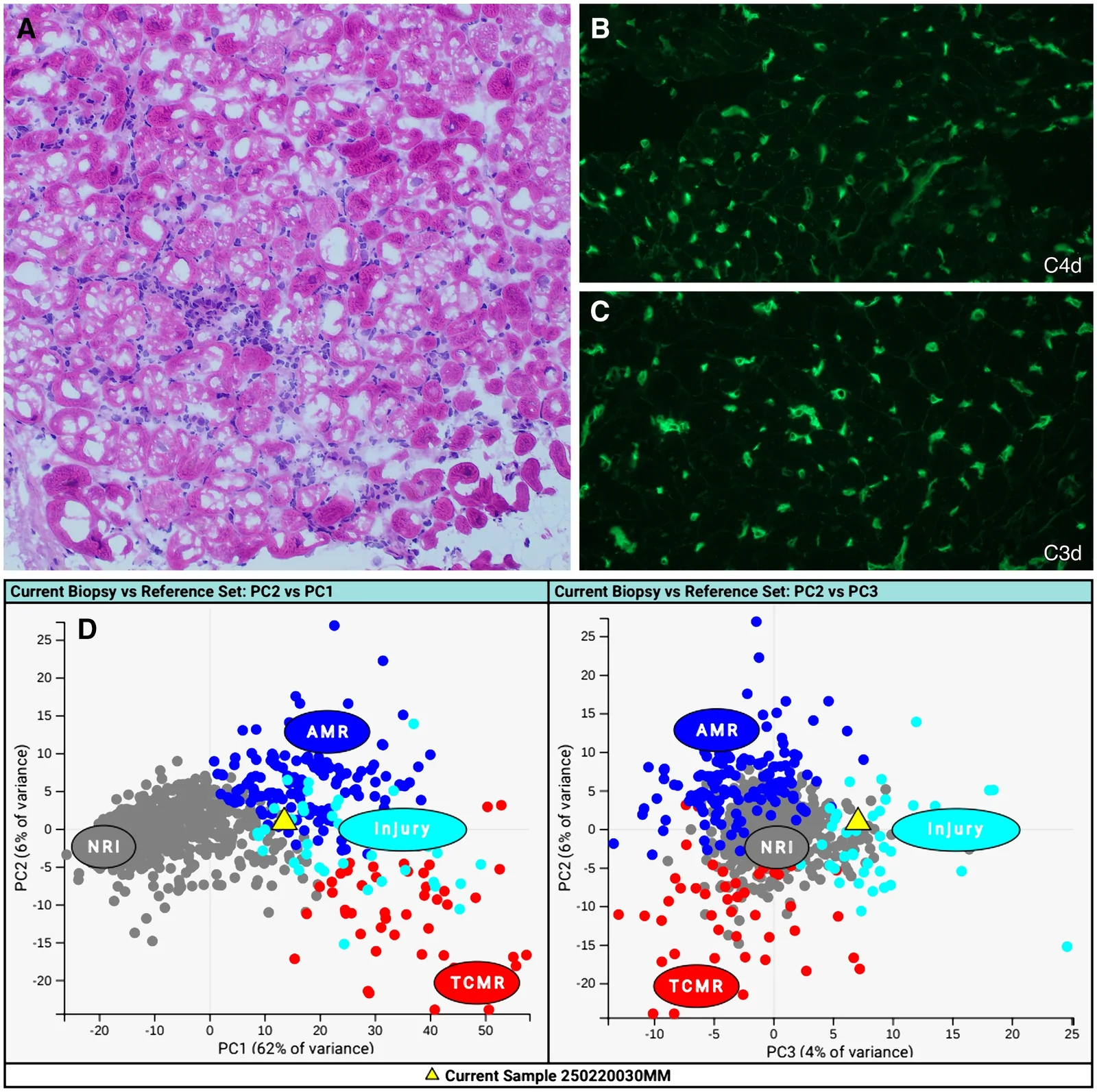
Endomyocardial biopsy and MMDx analysis. A, Hematoxylin and eosin. Endomyocardial biopsy showing 1R rejection. B, C4d immunofluorescence robustly staining capillaries of the myocardium. C, C3d immunofluorescence robustly staining capillaries of the myocardium. D, Results from MMDx. Scatter plots comparing the patient’s biopsy to a reference set using PC analysis. Colored clusters represent diagnostic categories: gray: NRI, blue: AMR, red: TCMR, and cyan: injury. Yellow triangle: Marks the position of the individual patient’s molecular profile within the reference data. Left scatter plot PC1 vs PC2: PC1 ranges from no rejection to rejection, while PC2 differentiates AMR (top) from TCMR (bottom). Right scatter plot PC2 vs PC3: PC2 differentiates AMR and TCMR, and PC3 ranges from no injury to injury. The current sample is near the AMR cluster but does not meet the AMR threshold. No evidence of TCMR. Moderate parenchymal injury. AMR, antibody-mediated rejection; MMDx, molecular microscope diagnostic system; NRI, no rejection/injury; PC, principal component; TCMR, T-cell–mediated rejection.

Echocardiogram showed a left ventricular ejection fraction of 60% and preserved right ventricular function (**Video S1A** and **B**, **SDC**). A sample from the second biopsy was sent for analysis using the molecular microscope diagnostic system (MMDx), which showed parenchymal injury but did not meet the threshold for AMR (Figure 1D). DSAs against HLA were not detected. A LABScreen Autoantibody Assay was performed on POD 10 to evaluate for non-HLA antibodies. Five antibodies were classified as positive with MFI cutoffs set at values ≤75% of the reference cohort. These included protein kinase C eta (PRKCH), C-X-C motif chemokine ligand 10 (CXCL10), glutathione S-transferase theta 1 (GSTT1), protein kinase C zeta (PRKCZ), and tumor necrosis factor-alpha (TNF-⍺). The patient was treated with intravenous methylprednisolone (1 g daily for 3 d), therapeutic plasma exchange on POD 13–15, and IVIg on POD 16 and 17.

After treatment, the MFIs of all 5 positive autoantibodies declined. GSTT1 and TNF-⍺ fell below the assay cutoff and were considered negative. PRKCH, CXCL10, and PRKCZ also decreased but remained above the assay cutoff (Table 1; Figure 2).

**TABLE 1.** Biopsy results and non-HLA antibody MFI

Non-HLA antibody	Cutoff (MFI)	Pretreatment		Posttreatment no. 1		Outpatient follow-up			
		POD 10		POD 18		POD 82		POD 152	
		Biopsy: ACR 2R; AMR (I+); C3d 2+; C4d 3+		Biopsy: ACR 1R; AMR (I+); C3d 1+; C4d 2+		Biopsy: ACR 1R; AMR (0); no C3d; no C4d		Biopsy: ACR 1R; AMR (0); no C3d; no C4d	
		MFI	ABC	MFI	ABC	MFI	ABC	MFI	ABC
PRKCH	767	4814	6.3	2560	3.3	1551	2.0	846	1.1
CXCL10	211	779	3.5	287	1.4	183	0.9	112	0.5
GSTT1	2609	7653	3.5	1933	0.7	216	0.1	161	0.1
PRKCZ	4066	6880	1.7	4315	1.1	5262	1.3	7332	1.8
TNF-α	2179	8908	2.4	2000	0.9	6035	2.8	6756	3.1

ABC was calculated as MFI divided by the assay-specific cutoff. The cutoff corresponds to 75% of the reference background values determined by screening >100 nontransplanted individuals. ABC ≥1 indicates values above the cutoff; ABC <1 indicates values below the cutoff.

ABC, above cutoff; ACR, acute cellular rejection; AMR, antibody-mediated rejection; CXCL10, C-X-C motif chemokine ligand 10; GSTT1, glutathione S-transferase theta 1; MFI, mean fluorescence intensity; POD, postoperative day; PRKCH, protein kinase C eta; PRKCZ, protein kinase C zeta; TNF-α, tumor necrosis factor-alpha.

**FIGURE 2.**
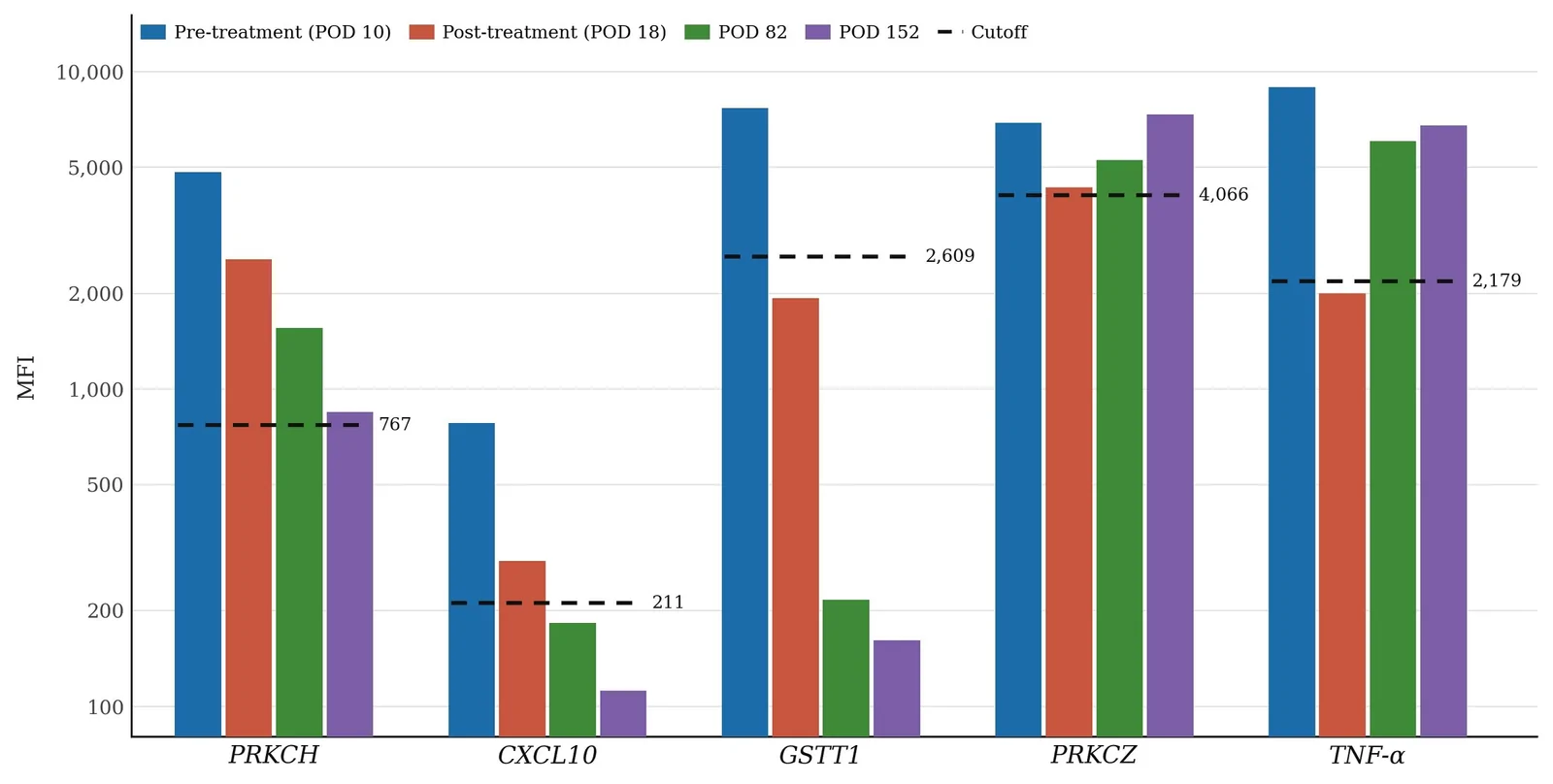
Non-HLA antibody trends. MFI values are shown for each non-HLA antibody across time points. Dashed lines indicate assay cutoff values, defined as 75% of the reference background (mean MFI of >100 in nonsensitized individuals). CXCL10, C-X-C motif chemokine ligand 10; GSTT1, glutathione S-transferase theta 1; MFI, mean fluorescence intensity; POD, postoperative day; PRKCH, protein kinase C eta; PRKCZ, protein kinase C zeta; TNF-α, tumor necrosis factor-alpha.

Follow-up EMB conducted on POD 18 demonstrated 1+ C3d and 2+ C4d. The patient received 3 additional plasma exchange sessions and IVIg. A subsequent biopsy on POD 29 revealed ACR 1R, with no C3d and 2+ C4d deposition.

The patient was discharged on POD 33 with prednisone, mycophenolate mofetil, and tacrolimus. The first 2 outpatient EMBs consistently showed ACR 1R, with no C3d and 1+ C4d.

Biopsies performed on POD 82 and POD 152 showed ACR 1R without evidence of C3d or C4d deposition. Echocardiogram demonstrated normal biventricular allograft function, and DSAs remained undetectable. MFI values of PRKCH, CXCL10, and GSTT1 continued to decrease over time. However, PRKCZ and TNF-α showed rebound increases (Table 1; Figure 2).

At 6 mo posttransplant, 1 episode of ACR 2R rejection occurred in the setting of missed immunosuppression during a rhinovirus infection. The episode responded to intravenous methylprednisolone. No further episodes of AMR were observed, and no DSAs were detected >1.1 y of follow-up.

## DISCUSSION

AMR has been associated with graft dysfunction and cardiac allograft vasculopathy. The established mechanism involves DSAs binding to HLA epitopes on donor endothelial cells, resulting in complement-mediated tissue injury.^1^ Approximately 40% of patients with pathology compatible with AMR have no detectable DSAs.^2,3^ To diagnose this condition, it is essential to consider potential causes of failure to detect pathogenic HLA-DSA. False-negative HLA-DSA can occur for 3 main reasons: limitations of the Luminex single-antigen bead assay, epitope-specific antibodies, and transient antibodies below the detection limit.^4,5^ In our patient, the rapid manifestation of AMR with negative virtual crossmatch and negative flow cytometry retrospective crossmatch, along with more than a year of follow-up and 18 tests with undetectable DSA (MFI <1000), makes a false-negative DSA result unlikely.

Histological features such as endothelial activation or swelling and macrophage adherence to the vascular endothelium have sensitivities of 63% and 30% for AMR, respectively.^6^ The reported sensitivity and specificity of C4d and C3d positivity for diagnosing AMR are 100% and 99%, respectively.^7^ The patient presented mixed rejection (ACR 2R and pAMR 1 [I+]), which has a reported prevalence of 7.8%.^8^

A significant increase in cytokines, including interleukin-6, interleukin-8, and complement activation (C3a), has been reported in the first weeks after LVAD implantation, but levels typically return to baseline by 6 wk.^9^ In spite of chronic driveline infection, our patient’s C-reactive protein level was <0.3 mg/dL 1 mo before the transplant, and pericapillary C3d and C4d deposits are not typically seen in nonspecific tissue injury. Given that pAMR 1 (I+) occurred within 2 wk after transplant, we did not order donor-derived cell-free DNA.

The Heart MMDx uses microarray technology to measure intragraft gene expression profiling. Rejection-associated transcript-based algorithms assess the probabilities of normal, AMR, T-cell–mediated rejection, and recent parenchymal injury.^10^ In the AMR discovery cohort, 83% of patients had detectable DSAs, 28% had pAMR 1 (I+), and 60% of those were molecularly inactive, like our patient.^11^

The Trifecta-Heart Study, was a investigator-initiated, prospective multi-center clinical trial designed to evaluate the correlation of donor-derived cell-free DNA with changes in genome-wide gene expression profiles using MMDx and central HLA antibody tests. A report of the Trifecta-Heart Study which evaluated DSAs-negative and DSAs-positive molecular AMR, demonstrated that gene expression profiles, including interferon-gamma-inducible and natural killer-expressed genes, did not differ significantly between the 2 conditions. Histologically diagnosed AMR was present in 1/3 of patients with molecular AMR.^12^ In our patient, MMDx was consistent with parenchymal injury but did not meet the threshold for AMR. Implementing this technique for real-time decision-making will require shorter turnaround times (case study turnaround time: 7 d).

The impact of non-HLA immunity on graft failure remains an active area of investigation.^13-15^ Testing of non-HLA antibodies has not been recommended in recent consensus statements, given the lack of evidence of a causal role in heart transplantation and the absence of standardized cutoffs.^16-18^ Our patient exhibited characteristics associated with non-HLA antibodies in previous studies, including an LVAD and a calculated panel reactive antibody exceeding 10%.^5,15,19,20^

Commercially available multiplex platforms for non-HLA testing include One Lambda LABScreen autoantibody assay (default test at our institution), which evaluates 39 autoantigens, and Immucor LIFECODES non-HLA autoantibody assay, which detects 60 distinct autoantigens. GSTT1, CXCL11, PRKCH, PRKCZ, vimentin, tubulin, actin, collagen, and myosin are among the 25 autoantigens detected on both platforms.^21^ Angiotensin II type 1 receptor antibodies are included in LIFECODES but not in LABScreen. Antibodies directed against major histocompatibility complex class I-related chain A are ordered as a separate test.^20^

In our case, 5 non-HLA antibodies were detected (GSTT1, CXCL10, PRKCH, PRKCZ, and TNF-⍺). GSTT1 has been associated with AMR and graft loss in renal transplantation.^22-24^ Yeo et al^25^ found that GSTT1 antibodies were associated with increased allograft rejection risk in heart and lung transplant patients, driven primarily by higher rates of cellular rejection in heart transplant patients. Increased levels of anti-CXCL11, which, like CXCL10, is a C-X-C motif chemokine receptor 3 ligand, have been reported during cardiac transplant rejection, and a potential autoregulatory role has been suggested.^26^ PRKCH is a key regulator of pre-B-cell receptor signaling and has been associated with transplant rejection in the presence of DSAs.^26^ PRKCZ antibodies have been detected with higher frequency among patients with AMR.^19,27^

Given the limited evidence supporting a causal role for these antibodies, we performed repeat testing to track their trajectories. Three antibodies (GSTT1, CXCL10, and PRKCH) showed a progressive decrease, while 2 (PRKCZ and TNF-⍺) exhibited a rebound pattern. We used the manufacturer-recommended 75% cutoff. We reanalyzed the posttransplant sample using a more specific 95% cutoff, and PRKCH, CXCL10, and TNF-α remained positive. The clinical and biological significance of these trajectories is unknown, but they may be useful for establishing a baseline in cases of recurrent episodes of DSA-negative AMR.

## CONCLUSIONS

In the setting of immunopathologic evidence of AMR within 2 wk after transplantation, with undetectable DSAs, we propose an integrated approach that may include evaluation of non-HLA antibodies.

In our patient, hemodynamic stability, preserved graft function, absence of progressive histologic features of AMR, gene expression profiling that did not meet AMR criteria, a shorter time frame of resolution of C3d, in comparison with C4d, and an initial decline in non-HLA antibody levels supported a management strategy without escalation to additional B-cell– or plasma cell–targeted therapies, such as rituximab or daratumumab.

## Supplementary Material



## References

[1] Mehta A DeFilippis Ersilia M Stehlik J. Contemporary antibody-mediated rejection in heart transplantation. JACC: Heart Failure. 2025;13:102614. 10.1016/j.jchf.2025.102614 PMC13537435 40848703

[2] Clerkin KJ Restaino SW Zorn E. The effect of timing and graft dysfunction on survival and cardiac allograft vasculopathy in antibody-mediated rejection. J Heart Lung Transplant. 2016;35:1059–1066. 10.1016/j.healun.2016.04.007 PMC5662939 27423693

[3] Cusi V Cardenas A Tada Y. Surveillance donor-specific antibody and pathologic antibody-mediated rejection testing in heart transplant patients in the contemporary era. J Heart Lung Transplant. 2025;44:1036–1049. 10.1016/j.healun.2025.01.019 PMC12206481 39914762

[4] Carey BS Boswijk K Mabrok M. A reliable method for avoiding false negative results with Luminex single antigen beads; evidence of the prozone effect. Transpl Immunol. 2016;37:23–27. 10.1016/j.trim.2016.04.002 27109036

[5] Filippone EJ Farber JL. Histologic antibody-mediated kidney allograft rejection in the absence of donor-specific HLA antibodies. Transplantation. 2021;105:e181–e190. 10.1097/TP.0000000000003797 33901113

[6] Hammond MEH Stehlik J Snow G; Utah Transplant Affiliated Hospitals (UTAH) Cardiac Transplant Program. Utility of histologic parameters in screening for antibody-mediated rejection of the cardiac allograft: a study of 3,170 biopsies. J Heart Lung Transplant. 2005;24:2015–2021. 10.1016/j.healun.2005.08.014 16364843

[7] Tan CD Sokos GG Pidwell DJ. Correlation of donor-specific antibodies, complement and its regulators with graft dysfunction in cardiac antibody-mediated rejection. Am J Transplant. 2009;9:2075–2084. 10.1111/j.1600-6143.2009.02748.x 19624562

[8] Kfoury AG Miller DV Snow GL. Mixed cellular and antibody-mediated rejection in heart transplantation: in-depth pathologic and clinical observations. J Heart Lung Transplant. 2016;35:335–341. 10.1016/j.healun.2015.10.016 26586489

[9] Corry DC DeLucia A 3rd Zhu H. Time course of cytokine release and complement activation after implantation of the HeartMate left ventricular assist device. ASAIO J. 1998;44:M347–M351. 10.1097/00002480-199809000-00005 9804450

[10] Halloran PF Madill-Thomsen KS. The molecular microscope diagnostic system: assessment of rejection and injury in heart transplant biopsies. Transplantation. 2022;107:27–44. 10.1097/TP.0000000000004323 36508644

[11] Loupy A Duong Van Huyen JP Hidalgo L. Gene expression profiling for the identification and classification of antibody-mediated heart rejection. Circulation. 2017;135:917–935. 10.1161/CIRCULATIONAHA.116.022907 28148598

[12] Madill-Thomsen KS Hidalgo LG Mackova M; Trifecta-Heart Study Group. Comparing DSA-negative and DSA-positive antibody-mediated rejection in heart transplants: results from the Trifecta-Heart study. J Heart Lung Transplant. 2026;45:1046–1057. 10.1016/j.healun.2026.03.004 41833593

[13] Reindl-Schwaighofer R Heinzel A Kainz A; iGeneTRAiN consortium. Contribution of non-HLA incompatibility between donor and recipient to kidney allograft survival: genome-wide analysis in a prospective cohort. Lancet. 2019;393:910–917. 10.1016/S0140-6736(18)32473-5 30773281

[14] Opelz G; Collaborative Transplant Study. Non-HLA transplantation immunity revealed by lymphocytotoxic antibodies. Lancet. 2005;365:1570–1576. 10.1016/S0140-6736(05)66458-6 15866311

[15] Oh GC Youn JC Cho HJ. Utility of non-HLA autoantibodies in predicting graft survival after heart transplantation. Transplantation. 2025;109:1449–1457. 10.1097/TP.0000000000005368 40128156

[16] Quintiliano A Agrawal A Zuccarelli M. Assessing reference ranges for non-HLA antibodies: comparative analysis of blood donors and kidney transplant candidates. Transpl Immunol. 2026;95:102364. 10.1016/j.trim.2026.102364 41692072

[17] Agrawal A Zuccarelli M Wakefield LL. Testing controls demonstrates a high prevalence of non-HLA antibody positivity using pre-defined cutoff threshold in a bead-based assay using healthy control population. Transpl Immunol. 2025;92:102265. 10.1016/j.trim.2025.102265 40680896

[18] Kobashigawa J Zuckermann A Zeevi A; Consensus Conference participants. Summary of the International Society for Heart and Lung Transplantation consensus conference on emerging understanding of antibodies and antibody-mediated rejection in heart transplantation. J Heart Lung Transplant. 2025;44:e1–e20. 10.1016/j.healun.2025.02.1690 40498433

[19] See SB Mantell BS Clerkin KJ. Profiling non-HLA antibody responses in antibody-mediated rejection following heart transplantation. Am J Transplant. 2020;20:2571–2580. 10.1111/ajt.15871 PMC8117249 32190967

[20] Panicker AJ Prokop LJ Hacke K. Outcome-based risk assessment of non-HLA antibodies in heart transplantation: a systematic review. J Heart Lung Transplant. 2024;43:1450–1467. 10.1016/j.healun.2024.05.012 38796046

[21] Oh I Song EY. Non-human leukocyte antigen antibodies in kidney transplantation: pathogenic mechanisms, detection methods, and clinical implications. Clin Transplant Res. 2025;39:214–229. 10.4285/ctr.25.0057 PMC12521850 40999815

[22] Aguilera I Alvarez-Marquez A Gentil MA. Anti-glutathione S-transferase T1 antibody-mediated rejection in C4d-positive renal allograft recipients. Nephrol Dial Transplant. 2008;23:2393–2398. 10.1093/ndt/gfm955 18308775

[23] Obrișcă B Leca N Chou-Wu E. Anti-GSTT1 antibodies and null genotype correlate with histological changes of antibody mediated rejection in kidney transplantation. Transpl Immunol. 2023;81:101943. 10.1016/j.trim.2023.101943 37866670

[24] Comoli P Cioni M Ray B. Anti-glutathione S-transferase theta 1 antibodies correlate with graft loss in non-sensitized pediatric kidney recipients. Front Med (Lausanne). 2022;9:1035400. 10.3389/fmed.2022.1035400 PMC9748144 36530923

[25] Yeo HJ Cho WH Kim HJ. Pre-existing non-human leukocyte antigen antibodies are associated with allograft rejection after thoracic transplantation. Transpl Immunol. 2023;77:101794. 10.1016/j.trim.2023.101794 36702359

[26] Butler CL Hickey MJ Jiang N. Discovery of non-HLA antibodies associated with cardiac allograft rejection and development and validation of a non-HLA antigen multiplex panel: from bench to bedside. Am J Transplant. 2020;20:2768–2780. 10.1111/ajt.15863 PMC7494540 32185871

[27] Sutherland SM Li L Sigdel TK. Protein microarrays identify antibodies to protein kinase Czeta that are associated with a greater risk of allograft loss in pediatric renal transplant recipients. Kidney Int. 2009;76:1277–1283. 10.1038/ki.2009.384 PMC4464694 19812540

