# Supplementary material for: Phenotyping Antibody-mediated Rejection Without Detectable Donor-specific Antibodies 8 d After Heart Transplantation: A Case Study
Source: Transplant Direct. 2026 Oct 21;12(11):e2016. doi: 10.1097/TXD.0000000000002016 (PMC13690437; doi:10.1097/TXD.0000000000002016)
Supplement: Supplementary file 1 [file txd-12-e2016-s001.pdf]

Table 1S. Pre-transplant cPRA details.

Serologic specificity	Allele specificity	MFI value*
B57, Bw4	B*57:03	1076
B57, Bw4	B*57:03	1041
DR4	DRB1*04:04	2401
DR4	DRB1*04:03	1846
DR4	DRB1*04:05	2021
DR4	DRB1*04:07	1410
DR4	DRB1*04:02	1747
DR4	DRB1*04:01	1188
DR4	DRB1*04:11	1025
DR4	DRB1*04:10	789
DR4	DRB1*04:06	199

*MFI ≥1000 considered positive and ≥4000 strongly positive by our HLA laboratory.
